# Crying

**Published:** 1884-03

**Authors:** 


14. Crying: Young children never cry if all is well. If an infant cries, it is suffering. Each mother should notice the different cries of her child, for they mean different things ; a cry from hunger is very different from a cry from hurt. A sticking pin causes a quick, instantaneous cry ; a string or fastening which is too tight causes a fret at first, gradually increasing as the blood accumulates. The hunger cry does not come on suddenly, for the little thing begins to turn its head or face about, or makes motions with the tongue or lips ; if it cannot find the breast it begins to make a noise, gets more and more impatient, and finally breaks out into a fierce, mad cry. The wisdom of the mother, then, should be called into requisition in deciding what cries mean, but in all cases attend to them ; in a young infant it often means that a change of position is needed, or that it is too warm or too cold or thirsty, and wants a drink of cold water. A good plan always is when a child is fretful, notice at once if the feet and hands are warm. If the children are regularly fed as advised elsewhere they will never cry from hunger, unless their food is not sufficiently nourishing. A tearless cry means pain or suffering. When tears are abundant it is the cry of anger, and should always be disregarded ; very young children will soon find out as to such crying, "It's no use knocking at the door any more."

A moaning cry always indicates suffering, and should never be neglected.

Breathing in a healthy child is regular, slow, easy, and full ; in proportion as it is different in any case there is disease.

### THE IDEAL.

I think the song that's sweetest
Is the one that's never sung;
That lies at the heart of the singer
Too grand for mortal tongue.
And sometimes in the silence
Between the day and night,
He fancies that its measures
Bid farewell to the light.

A picture that is fairer
Than all that have a part
Among the masterpieces
In the marble halls of art,
Is the one that haunts the painter
In all his golden dreams,
And to the painter only
A real picture seems.

The noblest, grandest poem
Lies not in blue and gold
Among the treasured volumes
That rosewood bookshelves hold;
But in bright, glowing visions,
It comes to the poet's brain,
And when he tries to grasp it
He finds his effort vain.

A fairy hand from dreamland
Beckons up here and there,
And when we strive to clasp it
It vanishes into air.
And thus our fair ideal
Floats always just before,
And we with longing spirits
Reach for it evermore.

